# Understanding materials challenges for rechargeable ion batteries with *in situ* transmission electron microscopy

**DOI:** 10.1038/ncomms15806

**Published:** 2017-08-25

**Authors:** Yifei Yuan, Khalil Amine, Jun Lu, Reza Shahbazian-Yassar

**Affiliations:** 1 Chemical Sciences and Engineering Division, Argonne National Laboratory, 9700S Cass Avenue, Argonne, Illinois 60439, USA; 2 Department of Mechanical and Industrial Engineering, University of Illinois at Chicago, Chicago, Illinois 60607, USA

## Abstract

An in-depth understanding of material behaviours under complex electrochemical environment is critical for the development of advanced materials for the next-generation rechargeable ion batteries. The dynamic conditions inside a working battery had not been intensively explored until the advent of various *in situ* characterization techniques. Real-time transmission electron microscopy of electrochemical reactions is one of the most significant breakthroughs poised to enable radical shift in our knowledge on how materials behave in the electrochemical environment. This review, therefore, summarizes the scientific discoveries enabled by *in situ* transmission electron microscopy, and specifically emphasizes the applicability of this technique to address the critical challenges in the rechargeable ion battery electrodes, electrolyte and their interfaces. New electrochemical systems such as lithium–oxygen, lithium–sulfur and sodium ion batteries are included, considering the rapidly increasing application of *in situ* transmission electron microscopy in these areas. A systematic comparison between lithium ion-based electrochemistry and sodium ion-based electrochemistry is also given in terms of their thermodynamic and kinetic differences. The effect of the electron beam on the validity of *in situ* observation is also covered. This review concludes by providing a renewed perspective for the future directions of *in situ* transmission electron microscopy in rechargeable ion batteries.

## Abstract (web summary)

*In situ* TEM is a powerful tool that helps to understand energy storage behaviors of various materials. This review summarizes the critical discoveries, enabled by *in situ* TEM, in rechargeable ion batteries, and foresees its bright future for extensive applications.

In spite of the widespread applications of rechargeable ion batteries, these devices still face various materials and interfacial challenges that exclude them from high power and high-performance applications. These problems are complicated because of the evolution of battery electrodes, electrolytes and solid electrolyte interphases (SEIs) during battery operation, which cannot be easily investigated under the required sealed working environment. Various methods such as X-ray and neutron scattering, Raman, cathodoluminescence and electrochemical impedance spectroscopy have been developed to characterize the material properties and behaviours in a battery. Among these techniques, transmission electron microscopy (TEM) stands out for high spatial/temporal resolution, superior sensitivity to inhomogeneity and versatile capabilities for a systematic study of material phases, compositions and electronic structures. Therefore, real-time TEM techniques (*in situ*) are emerging as a powerful tool in revealing the underlying mechanisms responsible for the shortcomings of rechargeable ion batteries such as gradual capacity fading during cycling, poor power supply at low temperatures, thermal runaway and overcharge instability^1^.

Since the inception of *in situ* TEM techniques for battery research in 2010 (ref. 2), the electron microscopy community has been able to make exciting contributions to the fields that shed light on the understanding of material dynamics during electrochemical reactions. Therefore, it is an opportune time to review the most original contributions and discuss further opportunities in this area. This work differentiates itself from previous reviews on the application of *in situ* TEM in battery research^3^,^4^,^5^ by focusing on the critical challenges in modern lithium ion batteries and beyond Li^+^ systems, as well as on the differences between these systems. Figure 1a–e illustrates a working rechargeable ion battery where the most significant issues existing in the anode, cathode and liquid/solid electrolyte can be addressed by proper *in situ* TEM techniques. Examples of battery challenges are dendrite growth, cathode oxygen release, anode phase transformation, crystal expansion and interfacial delamination. Figure 1f categorizes the battery materials that have been studied using *in situ* TEM based on the specific electrochemical problem/issue they represent for the electrochemical systems based on both Li^+^ and beyond Li^+^. The discussion is organized to highlight the most significant findings in charge storage mechanisms in the electrode, thermal stability and structural engineering of the electrode, as well as in liquid and solid electrolytes for both lithium ion systems and new battery chemistries. In addition, we discuss the side effect of the electron beam on the observed electrochemistry results, and to what extent the nanoscale discoveries by *in situ* TEM can be applied to real battery electrochemistry at bulk level. Finally, we have proposed some emerging research directions where *in situ* TEM can make significant contributions.

## *In situ* TEM for rechargeable ion batteries

This review aims to address the *in situ* TEM-enabled scientific discoveries associated with the electrode, electrode/electrolyte interface of rechargeable ion batteries. Important considerations in the battery electrode, namely the charge storage dynamics and kinetics, the compositional and structural engineering and the thermal stability analysis are discussed in details. The electrode/electrolyte interface discussion mainly covers the topics of SEI and Li dendrite growth, and solid-state electrolytes. Battery systems beyond Li^+^ are attracting more and more interest nowadays, where *in situ* TEM has shown great potential in analysing these systems. Related findings and achievements in the areas of rechargeable batteries with metallic lithium and rechargeable batteries with non-Li candidates are thus summarized. At last, the effect of the electron beam on *in situ* results and the data validity is discussed with emphasis on the open cell and the sealed liquid-cell designs.

### Electrode

Development of advanced electrode materials such as Mn-rich layered cathode and nanostructured Si anode is facing critical problems that demand in-depth understanding of electrochemical reactions. The scientific challenges include, but are not limited to, how cathodes experience thermal degradation with compromised battery safety, what is the charge storage mechanism for different materials, how Li dendrites evolve during Li (de)plating and why repetitive cycling leads to capacity fading. *In situ* TEM has been shown to be quite powerful in addressing these questions.

*Charge storage dynamics and kinetics*. Intercalation, alloying and conversion reactions are generally recognized as three mechanisms dominating Li^+^ storage in battery electrodes^6^. Typical intercalation electrodes possess intrinsic one-, two- or three-dimensional openings to facilitate Li^+^ transport without significant structural change^6^. The alloying mechanism proceeds by direct bonding between inserted Li^+^ and the host element A (A for Si, Ge, Sn, and so on) with formation of Li–A alloys^6^. The conversion reaction happens when Li ions are inserted into nanosized binary compounds as denoted by MX (M for transitional metals Fe, Co, Cu, and so on, and X for O, S, F, and so on), and results in reduction of M cations to M^0^ and formation of LiX (ref. 7). Generally, the three mechanisms direct charge storage of electrode materials so differently that the resultant electrode capacity, morphology and structure are sharply distinct. Extensive *in situ* TEM studies have been conducted to reveal the details of these charge storage mechanisms, which are discussed separately as following.

**Figure 2:**
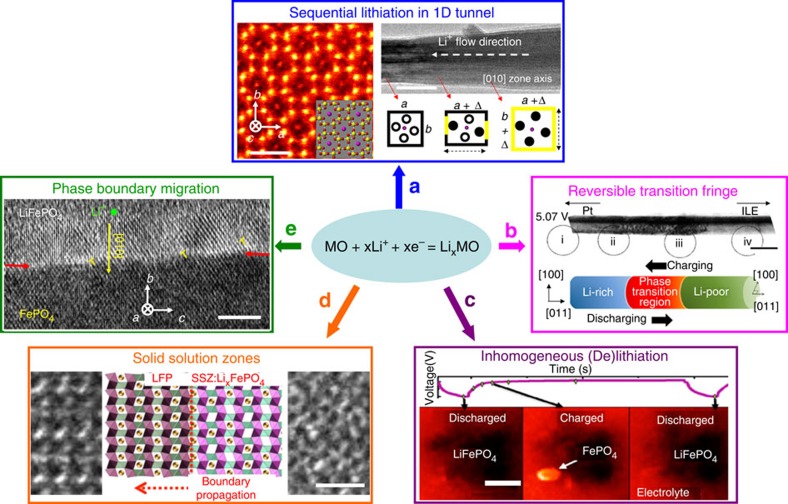
Examples of intercalation mechanisms revealed by *in situ* TEM. (**a**) Sequential Li^+^ filling inside α-MnO_2_ tunnels leads to crystal expansion asymmetry. The left scale bar , 1 nm, and the right bar, 50 nm. Adapted from ref. 8 (Copyright 2015 American Chemical Society). (**b**) Reversible movement of a phase transition region (red) between Li-rich and Li-poor phases during cycling of a single LiMn_2_O_4_ nanowire-based battery was captured. Scale bar, 200 nm. Adapted from ref. 9 (Copyright 2015 American Chemical Society). (**c**) Inhomogeneous (de)lithiation among LFP particles revealed by time-sequential energy-filtered TEM in an electrochemistry liquid cell. Scale bar, 200 nm. Reproduced from ref. 10 (Copyright 2014 American Chemical Society). (**d**) The movement of a solid solution zone was captured dynamically during delithiation of a LFP crystal. Scale bar, 1 nm. Adapted from ref. 11 (Copyright 2014 American Chemical Society). (**e**) The migration of a LFP/FP phase boundary was captured during lithiation with its migration direction identified as well. Scale bar, 5 nm. Adapted from ref. 12 (Copyright 2013 WILEY-VCH Verlag GmbH & Co. KGaA, Weinheim).

Ideally, intercalation electrodes should work without obvious structural degradation during ion insertion/extraction. Practically, however, localized structural instability has been reported mostly in the cathode side, leading to fast-capacity decay upon cycling. Therefore, it is necessary to understand the structural evolution details using *in situ* TEM possessing high spatial and temporal resolutions. In the case of MnO_2_ cathode featuring one-dimensional tunnelled structure, an asynchronous lattice expansion was found to be driven by sequential Jahn–Teller distortion of [MnO_6_] octahedral (Fig. 2a)^8^. This asynchronous expansion degrades the original tetragonal symmetry and causes tunnel instability that leads to low practical capacity of rechargeable Li/MnO_2_ batteries. The (dis)charge processes in LiMn_2_O_4_ cathode were found to exhibit a ‘fringe’ region featuring cubic–tetragonal transition (Fig. 2b), while no fracture or crack was found in LiMn_2_O_4_ nanowires^9^, suggesting possible structural modification and capacity retention via nanoscale engineering. Inhomogeneous (de)lithiation was revealed in LiFePO_4_ (LFP) nanoparticles using *in situ* liquid TEM (Fig. 2c)^10^, which greatly supplements other *in situ* TEM reports showing either solid solution zones (Fig. 2d)^11^ or two-phase (LFP/FP) boundary (Fig. 2e)^12^ in partially (de)lithiated LFP. These kinetic features could account for the poor cycling performance of LFP cathodes. The dynamic observation of structure degradation and phase boundary migration buried under electrode surfaces demonstrates the powerful capability of *in situ* TEM in detecting the localized reaction mechanisms that are otherwise difficult to study by other *in situ* or *ex situ* techniques. Inspired by such discoveries, the battery performance of these materials could be potentially improved by structural and compositional modification such as particle size reduction and trace elemental doping to reduce stress concentration and minimize structural degradation.

**Figure 3:**
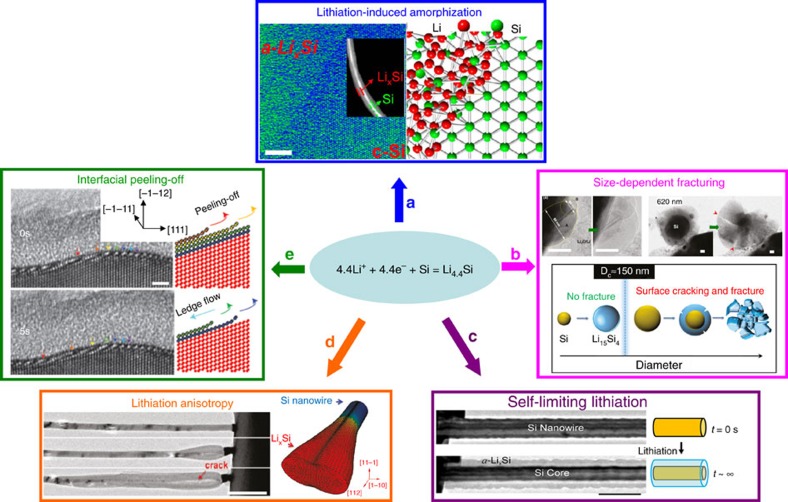
Alloying reactions between Li and Si studied by *in situ* TEM. (**a**) Evolution of the two-phase microstructure (Si–Li_*x*_Si core–shell) in one Si nanowire during lithiation. Scale bar, 5 nm. Adapted from ref. 13 (Copyright 2013 American Chemical Society). (**b**) Size-dependent fracturing of lithiated *c*-Si. Scale bars, 50 nm. Adapted from ref. 14 (Copyright 2012 American Chemical Society). (**c**) Self-limiting lithiation in single *c*-Si nanowire. Scale bar, 250 nm. Reproduced from ref. 15 (Copyright 2013 American Chemical Society). (**d**) Anisotropic expansion of lithiated *c*-Si nanowire leading to dumbbell morphology. Scale bar, 1 μm. Adapted from ref. 16 (Copyright 2011 American Chemical Society). (**e**) The lithiation of *c*-Si featuring lateral movement of ledges on the close-packed {111} atomic planes. Scale bar, 2 nm. Adapted from ref. 17 (Copyright 2012 Nature Publishing Group).

Among various alloying-based electrodes, silicon (Si) is of great interest because of its abundance in nature and high theoretical capacity of 4,200 mAh g^−1^ via the reaction: 4.4Li^+^+4.4+e^−^+Si=Li_4.4_Si. However, Si undergoes detrimental pulverization and capacity fading during repetitive cycling. Accordingly, intensive *in situ* TEM work has been done to explore the failure mechanisms during the Li–Si (de)alloying process (Fig. 3). To date, these discoveries include lithiation-induced amorphization^13^, size-sensitive fracturing^14^, self-limiting lithiation^15^, lithiation anisotropy^16^ and interfacial ledge-style lithiation^17^. Besides Si, elemental anodes such as Sn (ref. 18), Ge (ref. 19) and Au (ref. 20) were also studied by *in situ* TEM, where distinct Li (de)alloying behaviours are discovered. These inspiring findings have greatly improved the development of alloying electrodes, particularly Si-based anode. As such, their structural integrity and capacity retention are enhanced via surface and nanoscale engineering^21^,^22^,^23^,^24^,^25^.

**Figure 4:**
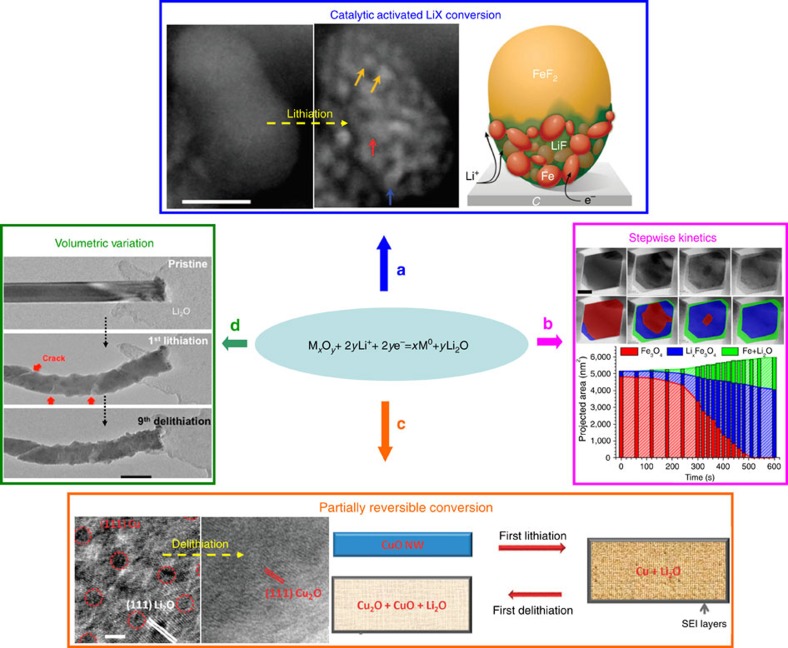
Mechanisms of conversion-based electrode explored by *in situ* TEM. (**a**) high resolution transmission electron microscopy (HRTEM) images and schematic showing the interconnected metal nanograins of several nanometres that improve the reactivity of LiX. Scale bar, 10 nm. Adapted from ref. 29 (Copyright 2012 Nature Publishing Group). (**b**) The identification of intermediate (intercalated) phases during the stepwise lithiation of Fe_3_O_4_ (ref. 30). Scale bar, 20 nm. (**c**) The low coulombic efficiency caused by the partially reversible conversion reaction between Cu^0^ and Cu^2+^. Scale bar, 2 nm. Adapted from ref. 34 (Copyright 2012 Royal Society of Chemistry). (**d**) Fracture formation in a RuO_2_ nanowire caused by volumetric variation during repetitive (de)lithiation. Scale bar, 100 nm. Adapted from ref. 32 (Copyright 2013 American Chemical Society).

Nanosized transitional metal oxides, sulfides and fluorides (MX) show reversible Li^+^ storage through a conversion reaction to M^0^ and LiX (ref. 7). Despite the decent capacity of these materials, several prominent problems/unknowns exist, such as the origins for high reactivity of LiX, the intermediate steps involving multiphase reactions, the low coulombic efficiency (CE) and the large overpotential and fast-capacity fading upon cycling^7^. High-magnification *in situ* TEM has captured the reversible formation of extremely small metallic nanograins M^0^ (2−3 nm) during the conversion of various MX phases^26^,^27^,^28^,^29^. Interestingly, these reduced M^0^ particles were found to be structurally connected with each other inside the LiX matrix, as illustrated in Fig. 4a, demonstrating the conductive Fe network after the conversion of FeF_2_ (ref. 29). The high surface area of the metallic nanograins and their network for fast electron conduction are critical to catalyse the reversible reaction involving LiX. Although the conversion reaction is thermodynamically favourable, nonequilibrium reaction kinetics were also revealed as evidenced by modified phase transition pathways and stepwise lithiation identified in nanosized Fe_3_O_4_ (Fig. 4b)^30^, MoO_3_ (ref. 31), MnO_2_ (ref. 8) and RuO_2_ (ref. 32). It is interesting to note that, even for materials containing the same metal cations (and anions), subtle differences in conversion kinetics exist. For example, while an intermediate insertion phase such as Li_*x*_Fe_3_O_4_ was detected prior to the conversion of nanosized Fe_3_O_4_ to Fe (ref. 30), the same was not observed for the conversion of nanosized Fe_2_O_3_ (ref. 33) or FeF_2_ (ref. 29) to Fe. Such differences in kinetics are possibly caused by the variation in ionic mobility of Li^+^, cations and anions that are confined within specific lattice frames. These indicate that in-depth investigations by *in situ* TEM are necessary to further understand the differences in metal oxides, sulfides and fluorides. The low CE is caused by partial reversibility of the conversion reaction, where metallic M^0^ nanograins are not always oxidized back to the original oxidation state but a lower valence state, as exampled by the conversion path via Cu^2+^O–Cu (discharge, 670 mAh g^−1^) and Cu–Cu^1+^_2_O (charge, 375 mAh g^−1^) in CuO (Fig. 4c)^34^ and other transitional metal oxide (TMOs) such as Fe_2_O_3_ (ref. 33) and Co_3_O_4_ (ref. 26). The cycling capacity fading is largely attributed to the volume change during the repetitive (de)lithiation that results in host fracturing, as recorded in various materials such as RuO_2_ (Fig. 4d)^32^ and Fe_2_O_3_ (ref. 33). The highly localized structural and morphological features in nanoscale during the conversion reaction requires the application of *in situ* TEM rather than any other collective *in situ* techniques such X-ray diffraction, Raman or X-ray photoemission spectroscopy. These *in situ* TEM findings strongly suggest the necessity of applying structural engineering and surface modification to improve the cycling stability of the TMO-based electrodes.

*Compositional/structural engineering*. Novel concepts have been implemented to improve the energy-storage performance of electrode materials via compositional design and structural engineering^35^. Although they are demonstrated to be effective, the underlying mechanisms improving the overall performance are poorly understood. Real-time TEM has been employed to study the working mechanisms of these concepts.

**Figure 5:**
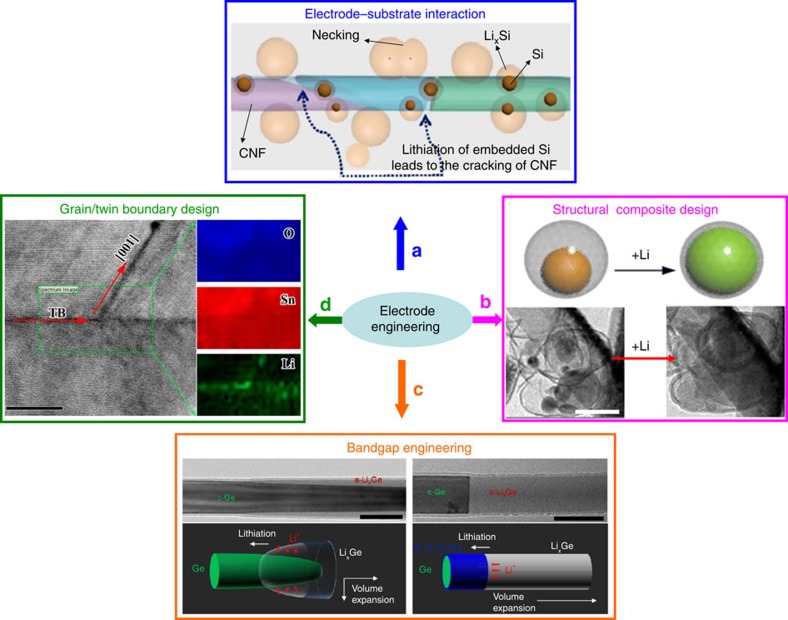
Mechanisms of compositional/structural engineering of electrode materials explored by *in situ* TEM. (**a**) Lithiation characteristics of Si particles attached to and embedded in the CNF. Adapted from ref. 36 (Copyright 2012 American Chemical Society). (**b**) Core–shell composite showing a free expanding Si core with stable carbon shell and SEI. Scale bar, 200 nm. Adapted from ref. 39 (Copyright 2012 American Chemical Society). (**c**) Lithiation kinetics in Ge core efficiently tuned by bandgap engineering using an extremely thin Si coating. Scale bars, 100 nm. Adapted from ref. 41 (Copyright 2013 American Chemical Society). (**d**) Twin boundary-assisted lithiation in SnO_2_. Scale bar, 10 nm. Reproduced from ref. 42 (Copyright 2015 American Chemical Society).

Silicon composited with carbon has been extensively studied to boost the electrical conductivity and mechanical flexibility of silicon anode. The spatial correlation between Si and carbon matrix and how various Si–C architectures (surface-attached Si, root-in-carbon Si) affect the (de)lithiation behaviours were well explored (Fig. 5a)^36^. Additional configurations have been further explored such as Si–carbon nanofibres^37^, Si–graphene^38^ and Si–C yolk-shell composites (Fig. 5b)^39^, as well as other composite systems beyond Si–C (CoS_2_–graphene^27^, NiO–graphene^35^ and Si–Sn (ref. 40)). Generally, two mechanisms were revealed by these studies, that is, the composites not only buffer the large volumetric variation of the loaded active materials and maintain stable SEI coatings on the surface, but also provide long-lasting e^−^/Li^+^ pathways during repetitive cycling. In addition, a novel bandgap engineering theory was demonstrated using Ge–Si composite (Fig. 5c)^41^, where a super thin (1−5 nm) Si coating on Ge surface efficiently changes the lithiation kinetics of Ge core. Next-generation batteries could potentially benefit from this idea by utilizing composite electrodes possessing less weight but better performance.

Crystal defects are conventionally expected to degrade battery performance, while *in situ* TEM has revealed positive roles of defects in enhancing battery reaction kinetics. For example, lithiation featuring stress-driven dislocation plasticity^2^ and twin boundary-assisted Li^+^ transport (Fig. 5d)^42^ were both discovered in crystalline SnO_2_ nanowires. Future battery work is expected to greatly benefit from these fundamental studies, assuming that the crystalline defects (dislocations, twins, stacking faults) could be reasonably generated and controlled during material synthesis.

*Thermal stability analysis*. With the increasing demand for densely packed lithium ion batteries for high-energy density applications, safety has become one of the major concerns. Localized thermal failure could result in chain reactions for large-scale thermal release and even explosion particularly in Co (Ni, Mn)-rich oxides at their charged states. Understanding the mechanisms of thermal failure is thus the prerequisite for effective improvement of battery safety.

With an *in situ* heating stage in TEM, the thermal decomposition of Li_*x*_Ni_0.8_Co_0.15_Al_0.05_O_2_ particles up to 450 °C was systematically studied and a layer to rock salt phase transition as well as surface porosity evolution associated with O release are dynamically recorded^43^. The origin of O release was further confirmed to be related to the reduction of Ni, which turns out to be less stable than other elements such as Mn and Co (ref. 44), indicating that the improved energy density of Ni-rich cathode materials can be compromised by its thermal instability. Real-time observation of O loss behaviours was also reported for LiCoO_2_, the well-known commercialized cathode material for lithium ion battery, where the O evolution shows facet-dependent feature and is correlated with local-phase transition (layer–spinel–rock salt) upon heating^45^. Future work should focus on engineering the electrode compositions and particle facets to reach a balance between synthesis cost, energy density and thermal stability.

### Electrochemistry interface

**Figure 6:**
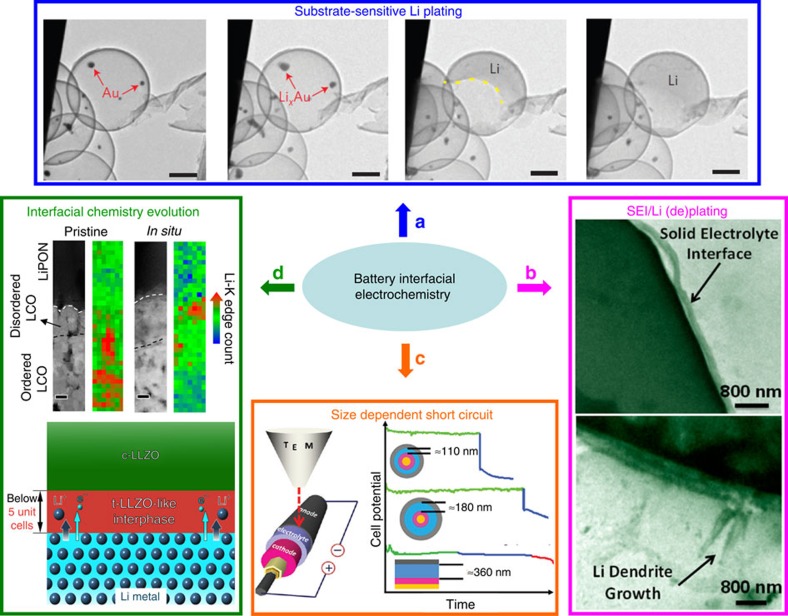
Li plating and SEI evolution in both liquid electrolyte and solid electrolyte explored by *in situ* TEM. (**a**) Preferred Li deposition on Au. Scale bars, 200 nm. Adapted from ref. 46 (Copyright 2016 Nature Publishing Group). (**b**) Nanoscale observation of SEI formation and Li dendrite growth on the electrode/liquid electrolyte interfaces^49^. Scale bars, 800 nm. (**c**) Self-discharge of a battery controlled by the thickness of electrolyte (LiPON). Adapted from ref. 54 (Copyright 2012 American Chemical Society). (**d**) Chemical and electronic structure evolution at the interfaces of LiCoO_2_/LiPON (top; adapted from ref. 55, Copyright 2016 American Chemical Society) and Li_(7−3*x*)_Al_*x*_La_3_Zr_2_O_12_/Li (bottom; reproduced from ref. 56, Copyright 2016 American Chemical Society) during cycling of thin-film solid-state batteries. Scale bar, 200 nm.

*SEI and Li dendrite growth*. Direct observation of the evolution of SEI and Li (de)plating on electrode/electrolyte interface with high spatial resolution is the prerequisite for understanding and improving interface-limited electrochemistry in batteries. Using *in situ* TEM, the substrate-sensitive Li plating behaviour (Fig. 6a)^46^ and the tunable Li plating and SEI stability via nanoscale interfacial engineering^47^ were both discovered, which could potentially guide the electrode design with stabilized metallic Li anode for better cycling durability.

Since the actual environment for SEI evolution and Li (de)plating typically contains liquid-based electrolytes, a sealed liquid cell has been increasingly utilized to mimic the authentic battery reaction environment^10^,^48^. Li dendrite growth and SEI formation/decomposition in a LiPF_6_/ethylene carbonate (EC)/diethyl carbonate (DEC) electrolyte was dynamically recorded at nanoscale resolution (Fig. 6b)^49^, where the kinetics of Li dendrite growth and dissolution are explored to explain the formation of ‘dead’ Li during cycling. The overpotential-dependent Li protrusion (root growth versus tip growth) was recently reported, where root-grown whiskers were seen to be highly unstable upon delithiation^50^. SEI formation kinetics were also found to be limited by electron transport as extensively confirmed^48^,^51^. In a LiPF_6_/EC/DMC liquid electrolyte, surprisingly, SEI formation is not uniform but in the shape of ‘dendrites’ similar to Li dendrites, and its growth is followed by Li plating^52^, implying the critical effect of electrolyte composition and electrode decomposition on Li plating. These *in situ* observations could help researchers to understand the kinetics of Li deposition and SEI growth and further to circumvent the related problems and battery failure.

*Solid-state electrolyte*. Compared to the conventional flammable liquid electrolyte in lithium ion batteries, solid-state electrolyte (SSE) is safe, nonflammable and compatible with metallic Li, and even allows more flexible configurations such as thin film and miniature batteries^53^. Currently, however, there are several critical issues not well understood, such as interfacial electric potential distribution, nanoscale (miniature battery) electrochemistry and Li^+^ transport property around the interface region. *In situ* TEM with analytical capability has been intensively applied to dynamically track interfacial Li^+^ diffusion/migration kinetics, providing in-depth understanding of the ionic transport mechanisms.

So far, two representative designs for the miniature solid-state batteries are proposed for *in situ* TEM study, that is, the single nanowire-based battery (Fig. 6c) and the thin-film battery (Fig. 6d). The former design enabled the discovery of size-sensitive self-discharge due to a short circuit caused by space-charge-limited electronic conduction^54^, thus providing useful metric guidelines for future miniature three-dimensional battery design. Nanoscale spectroscopic characterization across solid electrolyte/electrode interfaces (LiCoO_2_/LiPON and c-LLZO/Li) captured the existence of structurally disordered domains, unit cell-level phase transition and subsequent electronic structure change of transitional metals in the formed interphases (Fig. 6d)^55^,^56^. Within the similar thin-film battery design, the dynamic two-dimensional (2D) potential distribution caused by movement of Li^+^ near the cathode/SSE interface was quantified by *in situ* holography^57^. These *in situ* discoveries explain the commonly observed interfacial impedance in solid-state batteries. They also reveal the dynamic formation of electrode/SSE interfaces with factors affecting the interfacial stability and dimension, which thus provides a new perspective for designing Li/SSE/cathode assembly for next-generation batteries.

### Beyond lithium ion battery

*Rechargeable batteries with metallic lithium*. Current lithium ion batteries can hardly reach the high-energy density or longevity requirement to power electric vehicles with comparable performance to that of vehicles with internal combustion engines. Therefore, new electrochemical couples such as Li–O_2_ and Li–S are being targetedbecause of the much higher energy density^58^. Up to now, however, neither of them is commercialized due to practical challenges such as the large overpotential in the oxygen reduction reaction (ORR) and oxygen evolution reaction (OER), and the insulating property of Li_2_S and S (ref. 58). Until now, there are only a few *in situ* TEM publications focusing on the dynamic study of Li–S and Li–O_2_ electrochemistry.

The origin of the large charge overpotential in Li–O_2_ batteries has been ascribed to the e^−^ transport-limited charge kinetics based on *in situ* observation of Li_2_O_2_ decomposition using an open cell configuration^59^. Similar charge characteristics were also observed later from an organic liquid electrolyte, while the discharge process is surprisingly limited by Li^+^ diffusion, resulting in large overpotential during discharge as well^60^. These *in situ* reports demonstrate the importance of designing conductive porous cathode materials with ability to confine the dissolution of Li_2_O_2_ into the electrolytes as well as to maintain a good conductive path. For Li–S electrochemistry, the existence of S_8_/Li_2_S interface during discharge was captured in real time^61^, which was believed to prevent further Li^+^ diffusion into the bulk sulfur due to the insulating character of Li_2_S. Interestingly, a different theory regarding the observed flat Li_2_S/S interface was developed later to infer the electrically conductive property of such a Li_2_S/S_8_ interface^62^, which could thus encourage the modification/engineering of electrochemistry interfaces for improved battery performance. The application of protective layers carbon nanotube (CNT) on sulfur also demonstrated an efficient method to study electron beam-sensitive materials using TEM.

*Rechargeable batteries with non-Li candidates*. Alternative charge carriers are being extensively researched to replace Li, whose storage in the earth’s crust is too limited to meet the growing demand in the rechargeable battery market. Na^+^ is a very promising candidate because of its rich storage in the earth as well as its relatively light atomic weight^63^. Multivalent ions such as Mg^2+^, Ca^2+^ and Al^3+^ are also studied as promising candidates to replace Li^+^because of their high-energy density^64^. However, because of the variation in electronegativities and ionic sizes, the dynamic (dis)charge process exhibits distinct features compared to that in lithium ion batteries. Dynamic study in this area is still at the preliminary stage and current findings are frequently compared to that from the intensively studied Li^+^-based electrochemistry.

**Figure 7:**
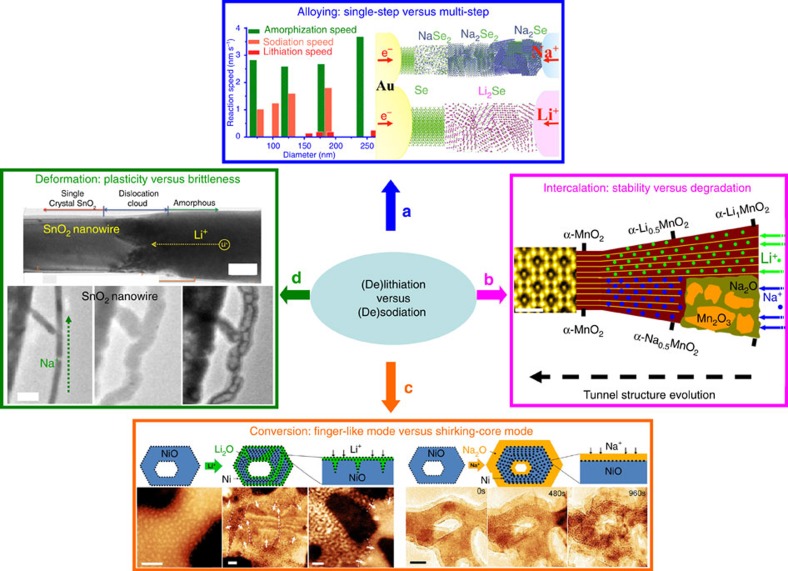
*In situ* TEM research comparing (de)sodiation and (de)lithiation in various electrode materials. (**a**–**c**) Lithiation versus sodiation in alloying (Se)-, intercalation (MnO_2_)- and conversion (NiO)-based electrode materials, respectively. Scale bars in **c**, 20nm. Panel **a** is reproduced from ref. 65 (Copyright 2016 American Chemical Society), **b** is adapted from ref. 68 (Copyright 2016 Elsevier Ltd.), **c** is adapted from ref. 69 (left) and ref. 70 (right) (Copyright 2015 American Chemical Society). (**d**) TEM images showing the lithiation-induced plasticity (top) and sodiation-induced softening/fracturing (bottom) in SnO_2_ nanowires. Scale bars, 200 nm. Adapted from ref. 2 (top; Copyright 2010 American Associate for the Advancement of Science) and ref. 73 (bottom; Copyright 2013 American Chemical Society).

It is of great significance to understand how Na^+^ and Li^+^ transport in battery electrodes differently and result in distinct material behaviours and battery performance. As an alloying anode, Se exhibits a multistep alloying mechanism with inserted Na^+^, which sharply differs from the direct single-step Se–Li alloying process (Fig. 7a)^65^. While similar intercalation mechanisms were observed for both lithiation and sodiation in 2D MoS_2_ (refs 66, 67), distinct Li^+^/Na^+^ intercalation behaviours were reported in α-MnO_2_ possessing one-dimensional tunnels, where the tunnels are more vulnerable during intercalation of Na^+^ than Li^+^ (Fig. 7b)^68^. For the conversion mechanism, the lithiation of NiO features a ‘finger-like’ heterogeneous reaction front^69^, while its sodiation is much delayed due to the ‘shrinking-core’ mode that thickens the Na_2_O surface layer and thus impedes further Na^+^ insertion (Fig. 7c)^70^. Distinct features during lithiation and sodiation were also found in other conversion-based materials such as CuO (refs 34, 71) and FeF_2_ (ref. 72). It is expected that the physical properties of the reaction interfaces should be responsible for the difference in reaction activities, structural stabilities and phase evolution during (de)lithiation and (de)sodiation, which apparently requires more fundamental study in the future.

Another notable difference between lithiation and sodiation is the mechanical response of materials considering the distinct sizes of Na^+^ (1.02 Å) and Li^+^ (0.76 Å). For example, the lithiation front in SnO_2_ nanowires features a dislocation-rich zone, making the lithiated SnO_2_ ductile and free of fracture, while Na^+^ interact with the host much stronger and results in a softened structure of poor plasticity, high Na^+^ diffusion barrier and disconnected Sn network, which largely decrease the sodiation kinetics (Fig. 7d)^2^,^73^. While such observation is reasonable, considering the much larger size of Na^+^ over Li^+^, counterintuitive observations were reported for the sodiation and lithiation in Se (ref. 65), Zn_4_Sb_3_ (refs 74, 75) and Co_9_S_8_ (refs 76, 77), where sodiation proceeds faster than lithiation over one order of magnitude. The improved reaction kinetics in sodiation of such materials might be ascribed to the high electrical conductivity of the various phases involved^74^. This theory is important in guiding the design and engineering of advanced battery electrodes with improved (dis)charge kinetics by controlling the phase transition pathways or modifying the conductivity of involved phases properly.

To date, there are few *in situ* TEM reports successfully capturing the insertion of multivalent ions into electrode materials. Ca^2+^ was first reported to be electrochemically inserted into single crystalline WO_3_ films in TEM, where an intercalation step (Ca_*x*_WO_3_) prior to conversion of WO_3_ into W and CaO was explicitly revealed at atomic scale^78^. In the case of Co_3_O_4_ nanocubes, Mg^2+^ insertion shows no sign of conversion reaction other than Mg plating, while Al^3+^ insertion is even more sluggish^79^. Such sluggish kinetics are largely determined by the inactive solid-state oxide layers (MgO and Al_2_O_3_) covering the surface of metallic anodes, as well as the large repulsive forces encountered by the inserted cations.

### Electron beam effect and electrochemistry in TEM

The energy of an incident electron during TEM operation ranges from tens to hundreds of KeVs, which can trigger side reactions in targeted materials and adversely affect the imaging process. The typical negative effects include atomic displacement and e-beam sputtering caused by elastic (electron–nucleus) scattering, and specimen heating/contamination/damage due to inelastic scattering^80^. Different materials exhibit distinct stability characteristics depending on the bonding strength between atoms, the mass of each element and the imaging condition. The extent by which the beam can affect any given material during the imaging process is a function of the beam-incident electron energy, dose rate and beam probe diameter. Such threshold values have been previously discussed and quantified^80^. Since a working battery requires the presence of electron transfer and the general usage of hydrocarbon-based materials that are sensitive to electron beam, the effects of an electron beam on *in situ* observation should be taken into serious consideration. These effects also vary based on different *in situ* TEM designs as categorized below.

**Figure 8:**
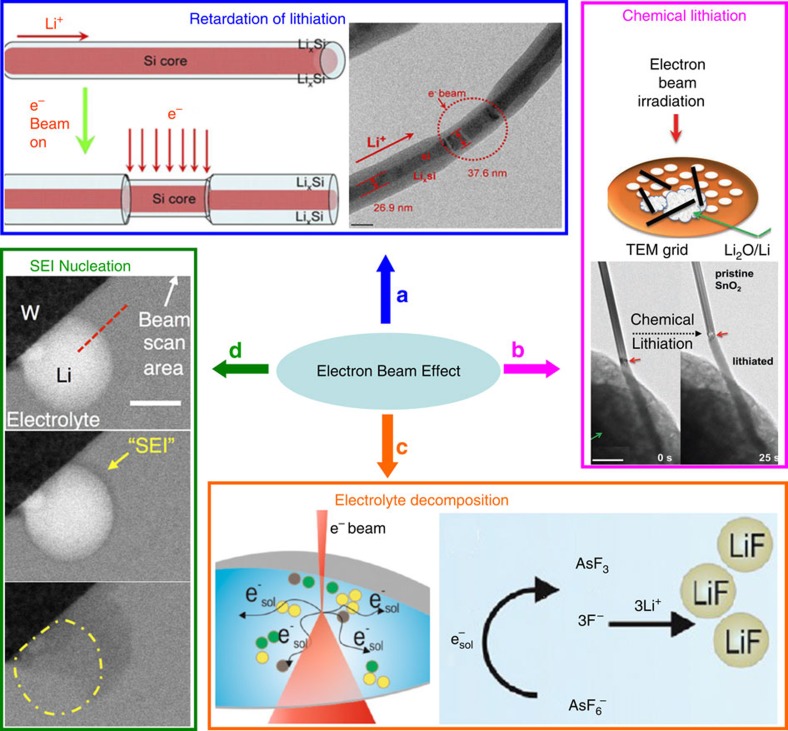
Electron beam effect on *in situ* TEM of battery electrochemistry. (**a**) Retardation of lithiation in a Si nanowire by electron beam. Scale bar, 50 nm. Adapted from ref. 3 (Copyright 2014 Materials Research Society). (**b**) Lithiation of a SnO_2_ nanowire driven by beam-induced Li_2_O decomposition. Scale bar, 500 nm. Adapted from ref. 5 (Copyright 2012 WILEY-VCH Verlag GmbH & Co. KGaA, Weinheim). (**c**) Schematics of beam-induced electrolyte decomposition as well as the proposed reduction mechanism of LiAsF_6_ in organic solvents. Adapted from ref. 82 (Copyright 2014 American Chemical Society). (**d**) Beam-induced SEI growth on the surface of Li deposit and at the expense of Li stripping. Scale bar, 300 nm. Adapted from ref. 48 (Copyright 2015 American Chemical Society).

*Beam effect on solid-state open cell design*. Within the solid-state open cell, the main concern is the stability of Li_2_O under electron beam and the subsequent effect on the observed battery electrochemistry. The electron beam was reported to either slow down the electrochemical lithiation (Fig. 8a)^3^ or initiate the chemical lithiation (Fig. 8b) by decomposing Li_2_O to release Li and lithiate nearby electrodes^5^. Fortunately, such decomposition can be alleviated by reducing the electron dosage below the safe dosage ∼1 A cm^–2^, and the chemical lithiation can thus be suppressed^5^. To further clarify the electron beam effect, contrast experiments have been done by controlling the electron energy and dosage, blocking the electron beam while not recording^75^ and referring to *ex situ* characterizations^19^. It is generally accepted that the material behaviours observed by *in situ* TEM using the open cell design could reflect the real battery electrochemistry in terms of reaction kinetics, structural change and chemical evolution^81^.

*Beam effect on sealed liquid-cell design*. Compared to the solid open cell, the *in situ* liquid-cell TEM is subject to more side reactions such as electrolyte breakdown^82^, bubble formation^83^ and nanoparticles’ precipitation/dissolution^82^,^84^. A range of (non)aqueous/Li salt electrolytes relevant to state-of-the-art Li–ion battery systems have been tested regarding their stability under electron beam^82^,^85^. In aqueous solutions, electron beam could generate various gases (bubbles) and free radicals that potentially affect the battery electrochemistry^85^. Browning and co-workers^82^ systematically studied electron beam radiolysis on nonaqueous electrolytes such as LiAsF_6_ in dioxolane (DOL), dimethyl carbonate (DMC) and DMC+EC, as well as LiTf in dimethylsulphoxide and LiPF_6_ in EC/DMC. A per-frame dose of 13.7 e^−^ nm^−2^ f is sufficient to cause breakdown of LiAsF_6_-based electrolytes with precipitation of LiF nanoparticles in the solution (Fig. 8c). LiPF_6_-based electrolyte is more stable as evidenced by the formation of fewer and smaller nanoparticles, while the LiTf-based electrolyte is very stable without any sign of breakdown. The observed stability variation among various electrolytes and the breakdown products and mechanisms agree with the *ex situ* battery tests, indicating the possibility of directly probing electrolyte stability using the *in situ* liquid scanning transmission electron microscopy technique. Following the decomposition of electrolytes (LiPF_6_/EC/DMC) under electron beam, other researchers also captured SEI nucleation and growth on Li deposit (Fig. 8d)^48^. The suitability of applying liquid TEM in real battery study is further demonstrated by obtaining the similar cyclic voltammetry (CV) curves with the beam on and off when testing nonaqueous electrolyte-based electrochemistry^10^.

## Perspectives for *in situ* TEM of rechargeable batteries

### Quantitative analysis to mimic *ex situ* battery testing

The quantification of nanoscale electrochemistry inside TEM is critical to correlate structural changes with the electrical output at various states of (dis)charge. While most *in situ* TEM research focuses on material analysis under constant voltages, better correlation with battery electrochemistry should be pursued. It is thus important to carry out real battery tests such as galvanostatic (dis)charge, CV, choronoamperometry and electrochemical impedance spectroscopy inside TEM. Recent reports have shown the possibility of measuring pA-level current flowing through a single nanowire-based battery^9^, observing small-scale SEI evolution and Li (de)plating in microfluidic TEM cells for galvanostatic^48^ and CV studies^86^. In extending the nanoscale findings to bulk-level battery electrochemistry, it is necessary to consider the impact of material interfaces and size effects on mass transfer and transport^87^. Future work should thus incorporate more details about the effect of cell dimension, geometrical electrode configurations and microfluidic conditions on the accuracy of electrochemistry quantification.

### Electrochemical evaluation under complex environment

With recent developments in the design of microfluidic and electromechanical chips and TEM column configurations, microscopists now can study electrochemical reactions in more complex environments. For instance, with the introduction of a thin-film heater within liquid cells, it would be quite interesting to study electrochemical reactions at various temperatures to understand the nature and composition of SEI growth as a function of temperature. These studies can also provide new understanding about the origins of thermal failure in rechargeable ion batteries. Another interesting study would be to introduce oxygen or water vapour in environmental TEM equipped with open cell battery holders to track the structural and chemical evolution of SSEs. This can help electrochemists to explore the degradation mechanisms accounting for the failure of solid-state batteries in ambient atmosphere. Including force measurement features in the electrochemical holders can be used to quantitatively explore the mechanical degradation in flexible batteries. Ion irradiation in TEM during electrochemical reactions can help us gain a better understanding of the working environment of delicate batteries widely used in aerospace and nuclear environments. These environments are rich in intensive ion flux, which can potentially result in ionization, atomic displacement, impurity production and energy release^88^.

### Electrochemistry studies with multiprobes

With the advent of new detection and imaging probes in TEMs, innovative windows of opportunities can be opened towards understanding the underlying mechanisms of complex electrochemical reactions. A direct observation of charge distribution under battery-operating conditions is important to assess interior potential distribution. Off-axis electron holography has been recently incorporated with *in situ* TEM to image the charge distribution in Ge nanowire during normal (de)lithiation^89^. Inspired by this design, future work can focus on exploring more about electrochemical dynamics that involve ion insertion and electron transport under battery-operating condition. The development of ultrahigh-speed cameras (up to 1,600 fps)^90^ is necessary considering the beam-sensitive nature of many battery components such as lithium and sodium salts, carbon-based materials and the liquid electrolyte. Imaging under even higher temporal resolution of nanoseconds is also necessary if one desires to visualize the transient atomic motions such as the breaking and formation of chemical bonds to understand the ultrafast structural dynamics^91^,^92^. This is also suitable for TEM studies of dynamic events under extremely cyclic/oscillatory conditions. Such high time resolution can be achieved via utilization of a pulsed electron beam, and this technique is ultimately limited by the ability of generating enough electron flux from the gun source^90^.

In addition to electrons, signals such as photons and secondary ions can also be generated in TEM. The capability of introducing light (laser) and simultaneously collecting the excited photons from targeted materials has been demonstrated previously for both Raman and cathodoluminescence spectroscopies^93^,^94^. Applying such techniques to battery systems, one can expect the real-time monitoring of battery temperature, chemical and compositional evolution and defect localization during the battery cycling inside TEM. Secondary ion mass spectroscopy has been developed to dynamically study electrochemistry interfaces buried in liquid electrolyte due to its sensitivity to the surface composition, molecular structure and elemental (especially for H and Li) distribution^95^. The previous work of synergizing focused ion beam (FIB) (for incident ions) and secondary ion mass spectroscopy (for secondary ion detection) within TEM^96^ has enlightened the possible incorporation of this technique for real-time battery study using *in situ* liquid-cell TEM, which can provide systematic information regarding interfacial dynamics and kinetics. However, the introduction of multistimuli and multiprobes into the narrow gap between the two pole pieces within TEM can potentially degrade the imaging resolution and increase the chance of cross-contamination, which will demand a careful design of the hardware configuration.

### Pushing towards high resolutions

One problem plaguing the *in situ* TEM community is the reduced spatial/energy resolution due to presence of membranes for microfluidic devices, thick liquid electrolytes or various stimuli along the beam path, which largely decrease the accuracy of imaging or spectroscopy analyses. In particular, the spatial resolution of the liquid cell falls far behind that of traditional imaging, not to mention the elemental and electronic structure sensitivity^97^. Therefore, future work should aim to increase the spatial resolution and the analytical ability of *in situ* TEM by reducing the liquid thickness along the electron path, replacing the thick SiN windows with thinner layers^98^, and engineering the pathway of characteristic X-rays travelling to the detector^99^. Another promising method recently developed for high-resolution imaging through liquid electrolyte is the use of atomically thin graphene to encapsulate a limited amount of liquid and form a sealed environment for *in situ* observation^100^,^101^. This strategy greatly reduces inelastic electron scattering and guarantees atomic-level imaging through the liquid. However, the thinner thicknesses mean that one should also consider the effect of confined volumes^87^ on the electrochemical reactions and how such reactions differ with the ones occurring at larger length scales.

### *In situ* TEM for future battery chemistries

With everyday progress in the field of *in situ* TEM, it is becoming important to tackle some of the scientific challenges that new battery chemistries are facing. For example, the study of Li–O_2_ batteries using *in situ* liquid TEM is very difficult because of the practical difficulty of introducing O_2_ in the TEM chamber. This complicated system, however, was simplified by flowing an O_2_-rich electrolyte into the liquid cell in TEM^60^. This opens up the idea for exploring the ORR and OER in subnanoscale. It would be of great interest to further investigate the charge/discharge reactions in Na–O_2_ and Zn–O_2_ batteries that have high chance of major breakthroughs. Another interesting chemistry would be the high-voltage aqueous electrolyte Li–ion batteries that have been realized by the ‘water-in-salt’ concept^102^. *In situ* liquid TEM experiments can probably replace the currently used organic electrolyte with aqueous electrolytes. This can ease the operational challenges of organic-based liquid electrolytes such as carbon contamination, liquid flow clogging and H_2_O-sensitive organic electrolyte. The study of multivalent batteries can be pursued with a liquid-cell TEM technique and with a proper choice of electrolytes and electrodes.

In addition to several promising directions to be further explored by *in situ* TEM, one should realize the limitations of this technique. These include the requirement of delicate specimens with extremely thin thicknesses, minimized contamination and maximized stability, as well as the effect of confined volumes on the electrochemical reactions. With these considerations, it is necessary to combine other complementary methods (for example, *in situ* X-ray scattering and Raman spectroscopy) with *in situ* TEM to enable a more comprehensive evaluation of dynamic electrochemical reactions.

To conclude, we have summarized the current scientific discoveries in rechargeable batteries (Li–ion, Li–O_2_, Li–S and Na–ion batteries) that have been realized by *in situ* TEM. Compared to other *in situ* techniques, TEM enables direct visualization and spectroscopy of the electrochemical processes at subnanoscales. The sensitivity of TEM in capturing localized information and inhomogeneity also complements other *in situ* methods sensitive to bulk information. Significant findings include, but are not limited to, the positive role of crystal defects in charge transport, anisotropic lithiation, phase boundary migration kinetics during cycling, SEI and metallic dendrite evolution in liquid electrolytes, and the key factors limiting the ORR and OER kinetics in metal–air batteries. We foresee that *in situ* TEM holds great potential to further advance our fundamental knowledge of nanoscale electrochemistry events in rechargeable ion batteries. Along these lines, multidimensional and cross-disciplinary efforts from electrochemists, microscopists and nanofabrication scientists are needed to address the current battery challenges and pave the roadmap for new discoveries in this field. Some of the future directions can encompass (1) performing quantitative electrochemistry at nanoscale; (2) advancing multiprobe analysis during nanoscale electrochemistry; (3) developing new stimuli to study batteries under nonequilibrium environments; (4) enhancing the image resolutions; and (5) exploring the challenges of new battery chemistries beyond Li–ion batteries.

## Additional information

**How to cite this article:** Yuan, Y. *et al*. Understanding materials challenges for rechargeable ion batteries with *in situ* transmission electron microscopy. *Nat. Commun.* **8,** 15806 doi: 10.1038/ncomms15806 (2017).

**Publisher’s note:** Springer Nature remains neutral with regard to jurisdictional claims in published maps and institutional affiliations.

## Figures and Tables

**Figure 1:**
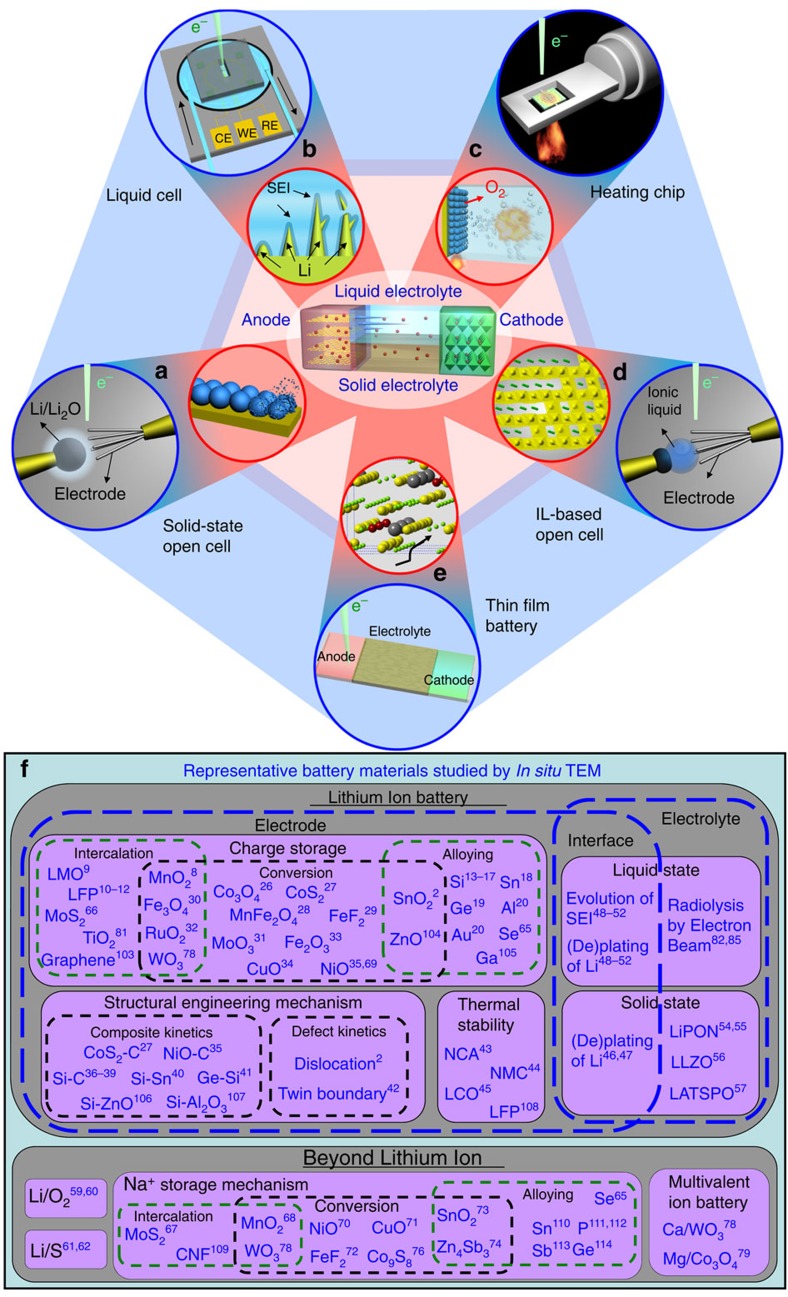
Various battery challenges and battery materials investigated by *in situ* TEM. (**a**–**e**) A working rechargeable ion battery (centre schematic) has many problems/challenges existing in the cathode, anode and liquid/solid electrolyte (inner circle), where each case is studied by a specific *in situ* TEM technique (outer circle). (**a**) A solid-state open cell exploring the structure failure (volume change, and so on) in anode. This design allows high spatial resolution imaging, but its point-contact geometry is different from the real battery environment flooded with liquid electrolytes. (**b**) A sealed liquid-cell investigating SEI and Li dendrites’ evolution at the electrolyte/electrode interface. This design suffers from low spatial resolution, but it is a better match to the practical batteries. (**c**) An *in situ* heating stage analysing the thermal stability of metal oxide-based cathode, where surface degradation with O_2_ release and thermal runaway is the targeted problem. (**d**) An ionic liquid-based open cell studying the phase transition in metal oxide-based cathode, where detrimental phase transitions plague the overall performance. (**e**) A nanoscale thin-film battery studying solid-state electrolytes, where low ionic diffusivity and interface instability are the targeted problems. (**f**) Representative battery materials studied by *in situ* TEM^2^^,^^8^,^9^,^10^,^11^,^12^,^13^,^14^,^15^,^16^,^17^,^18^,^19^,^20^^,^^26^,^27^,^28^,^29^,^30^,^31^,^32^,^33^,^34^,^35^,^36^,^37^,^38^,^39^,^40^,^41^,^42^,^43^,^44^,^45^,^46^,^47^,^48^,^49^,^50^,^51^,^52^^,^^54^,^55^,^56^,^57^^,^^59^,^60^,^61^,^62^^,^^65^,^66^,^67^,^68^,^69^,^70^,^71^,^72^,^73^,^74^,^75^,^76^,^77^,^78^,^79^^,^^82^^,^^85^^,^^103^,^104^,^105^,^106^,^107^,^108^,^109^,^110^,^111^,^112^,^113^,^114^. NCA, NMC, LFP, LMO and LCO stand for cathodes based on Ni-Co-Al-O, Ni-Mn-Co-O, LiFePO_4_, Li-Mn-O and Li-Co-O, respectively. LiPON, LLZO and LATSPO stand for solid-state electrolytes based on Li-P-O-N, Li-La-Zr-O and Li-Al-Ti-Si-P-O, respectively. For A–B expression, A represents the core component and B represents the shell or substrate component. CNF represents carbon nanofibres.
